# Pre-hospital and retrieval medicine in Scotland: a retrospective cohort study of the workload and outcomes of the emergency medical retrieval service in the first decade of national coverage

**DOI:** 10.1186/s13049-023-01109-6

**Published:** 2023-08-22

**Authors:** Ryan D McHenry, Christopher EJ Moultrie, Andrew J Cadamy, Alasdair R Corfield, Daniel F Mackay, Jill P Pell

**Affiliations:** 1 ScotSTAR, Scottish Ambulance Service, Hangar B, 180 Abbotsinch Road, Paisley, PA3 2RY UK; 2 School of Health and Wellbeing, University of Glasgow, 1 Lilybank Gardens, Glasgow, G12 8RZ UK

**Keywords:** Retrieval Medicine, Pre-Hospital Emergency Medicine, Mortality, Outcomes, Geospatial modelling

## Abstract

**Background:**

The Emergency Medical Retrieval Service (EMRS) has provided national pre-hospital critical care and aeromedical retrieval in Scotland since 2010. This study investigates trends in the service and patients attended over the last decade; and factors associated with clinical deterioration and pre-hospital death.

**Methods:**

A retrospective cohort study was conducted of all service taskings over ten years (2011–2020 inclusive). The EMRS electronic database provided data on location, sociodemographic factors, diagnoses, physiological measurements, clinical management, and pre-hospital deaths. Binary logistic regression models were used to determine change in physiology in pre-hospital care, and factors associated with pre-hospital death. Geospatial modelling, using road and air travel time models, was used to explore transfer times.

**Results:**

EMRS received 8,069 taskings over the study period, of which 2,748 retrieval and 3,633 pre-hospital critical care missions resulted in patient contact. EMRS was more commonly dispatched to socioeconomically deprived areas for pre-hospital critical care incidents (Spearman’s rank correlation, r(8)=-0.75, p = 0.01). In multivariate analysis, systolic blood pressure < 90mmHg, respiratory rate < 6/min or > 30/min, and Glasgow Coma Score ≤ 14 were associated with pre-hospital mortality independent of demographic factors. Geospatial modelling suggested that aeromedical retrieval reduced the mean time to a critical care unit by 1 h 46 min compared with road/ferry transportation.

**Conclusion:**

EMRS continues to develop, delivering Pre-Hospital and Retrieval Medicine across Scotland and may have a role in addressing health inequalities, including socioeconomic deprivation and geographic isolation. Age, specific distances from care, and abnormal physiology are associated with death in pre-hospital critical care.

**Supplementary Information:**

The online version contains supplementary material available at 10.1186/s13049-023-01109-6.

## Introduction

The Emergency Medical Retrieval Service (EMRS) was established in 2004 to support retrieval of critically unwell patients from Scottish rural hospitals [1, 2]. Initially operating in the West of Scotland from a Glasgow base, the service expanded to national coverage in October 2010, and a second base in Aberdeen opened in April 2019. Since 2014, EMRS has operated within the Scottish Specialist Transport and Retrieval (ScotSTAR) division of the Scottish Ambulance Service (SAS), which also includes specialist neonatal and paediatric teams facilitating remote and rural retrieval, and inter-hospital transfer, for these patient groups. EMRS works alongside the SAS Air Ambulance Service, which provides paramedic-led aeromedical retrieval, continuing operations established in 1933 [3].

Scotland covers 77,910 km², with 93 inhabited offshore islands. 6% of the population, 316,666 people, live in remote rural locations [4]. EMRS services are delivered by road, using dedicated response vehicles; or by air, primarily using SAS rotary or fixed-wing aircraft, with assistance from Scotland’s Charity Air Ambulance (SCAA) and Search and Rescue (SAR) aircraft when operationally required.

EMRS has evolved to facilitate both retrieval of critically unwell patients from remote and rural healthcare facilities; and provide pre-hospital critical care at the site of major injury, critical illness, and major incident. It responds with a two-clinician team, comprised of a Pre-Hospital and Retrieval Medicine Consultant and either a Retrieval Practitioner (from a paramedic or nursing background) or medical Speciality Trainee [5]. Tasking is facilitated by the SAS, with referrals from remote and rural clinicians coordinated through the Specialist Services Desk, which also supports transport logistics. In October 2012, a Trauma Desk was introduced, providing clinician-led dispatch for pre-hospital critical care taskings [6]. From its inception, EMRS has provided retrieval 24 h a day. While occasional ad-hoc pre-hospital critical care was available prior, from 2010 a funded service with a trauma-focussed remit has been available for immediate tasking during resident hours of 07.00-18.00 h (in Glasgow later extended to 23.00 h), and outside of these times to major incidents and prolonged entrapment. EMRS now covers healthcare sites with a range of capabilities, from single-clinician GP and nurse-led facilities, to rural and district general hospitals with the ability to institute initial critical care interventions. As such, EMRS interventions range from initial resuscitation, to direct initiation of critical care interventions, and continuation during transport of interventions commenced at a referring centre.

In this paper, we aim to update previous work describing the nature of the service [1, 7], illustrate the changing face of Pre-Hospital and Retrieval Medicine, determine factors associated with use and outcomes of the service, and share lessons that may be useful in the growth and development of similar services internationally.

## Methods

### Data collection

The national EMRS database comprises the electronic transcript of contemporaneous paper medical notes input by service clinicians, initially to a Microsoft Access database (Microsoft Corporation, Redmond, USA), transitioning in 2018 to a custom database (Nugensis Ltd., Glasgow, Scotland).

A retrospective cohort study was conducted of all EMRS taskings over the first 10 full calendar years as a national service (January 2011 – December 2020 inclusive), including those where EMRS was stood down prior to patient contact. Those where the referring team consulted the service only for telemedical advice were excluded from analysis.

The nature and extent of data collection has changed over time but, for the duration of this study, the minimum dataset included age, location, physiological parameters, interventions, diagnosis, pre-hospital mortality, free text comments on nature of mission, transport, and destination. Free text comments were searched for mechanism of injury in pre-hospital critical care missions. Diagnoses were mapped against principal diagnosis codes using the International Classification of Diseases, 10th Revision (ICD-10) [8].

### Statistical analyses

The cohort was stratified by retrieval versus pre-hospital critical care taskings. Cohort characteristics were summarised using median and range for continuous variables and frequency and percentage for categorical. The primary outcome was all-cause, pre-hospital death. Binary logistic regression analysis was used to determine sociodemographic (age, sex), service (distance from EMRS base) and physiological (systolic blood pressure, Glasgow Coma Score, and respiratory rate) factors associated with pre-hospital death. Multiple imputation, using missing at random assumptions, was used to manage missing data [9], reporting the pooled effect for regression models from five imputed data sets. The secondary outcomes were abnormal physiological observations (systolic blood pressure, Glasgow Coma Score, respiratory rate). Separate overlap binary logistic regression analyses were used to determine the factors associated with each of these outcomes between team arrival and two subsequent stages of EMRS care with end-points at team departure from patient location and arrival at destination hospital.

### Geographic modelling and analyses

Frequency of referrals were stratified by referring centre. The coordinates of pre-hospital critical care missions were obtained from the global positioning systems on board ambulance transport assets and used to categorise missions by Scottish Data Zone (mean population 784). Each Data Zone is ranked using the Scottish Index of Multiple Deprivation (SIMD), an area-based measure of socioeconomic deprivation derived from aggregate data across seven domains [10]. Spearman’s rank test was used to determine the correlation between the frequency of missions and SIMD decile.

Geospatial modelling of rotary aircraft travel time isochrones from each EMRS base, based on a cruising speed of 120 knots plus time for loading and unloading was used to determine the areas of Scotland where EMRS provided fastest access to the initiation of critical care. Areas where access by road to hospitals providing facilities to initiate critical care (defined as the presence of an Emergency Department and Anaesthetic capability) was equal to, or faster than, these aeromedical times, were subtracted from the isochrone map. The population-weighted centroids of data zones that fall within this area were used to calculate the approximate population within this area.

Modelled time from patient location to critical care unit (defined as the Scottish critical care unit reachable in the shortest time from each location) was used as a proxy measure of geographic isolation. Reflecting conventional land transport, time to critical care unit by road and ferry was modelled to each receiving centre using real-world travel data (Travel Time, *Isochrone API*, London; 2021). Additionally, the current configuration of aeromedical transport in Scotland was modelled and incorporated into a unified model of both road and aeromedical transport. After the methods by Emerson et al. [11], aeromedical time was modelled (using the same assumptions as above) for rotary aircraft, deployed from current Scottish aeromedical bases, to patient location, and then to major hospitals with helicopter landing sites and critical care units, resulting in ellipses.

Patients were not involved in study design. Reporting was aligned to STROBE guidelines [12]. Analyses were conducted using sf, traveltime, mice and stats packages in R [9, 13–15].

## Results

EMRS received 8,069 taskings between 1st January 2011 and 31st December 2020 inclusive. Of these, 5,261 (65.2%) were for pre-hospital critical care at the site of critical illness or injury, and 2,808 (34.8%) were for retrieval of critically ill patients from remote and rural healthcare facilities. Retrieval taskings per year remained largely stable across the study period (range 234–385; median 278). In contrast, pre-hospital critical care mission taskings increased more than twenty-fold, from 40 taskings in 2011 to a maximum of 927 in 2019 (Supplementary Fig. 1).

### Retrieval

Of the 2,514 patients referred for retrieval for whom sex was recorded, 1,060 (42.2%) were female. The median age of patients referred was 60 (range 1–96) years. The most common diagnostic groups for patients referred for retrieval were injury & poisoning (22.5%), and respiratory (20.7%) or circulatory (18.5%) diseases (Table 1).

**Table 1:** Characteristics of Emergency Medical Retrieval Service patients stratified by retrieval tasks and pre-hospital critical care attendances

	Retrieval n = 2,808	Pre-hospital critical care n = 3,633
	Median (Range)	
**Age (years)**	60 (1–96)	42 (0–97)
Missing	133	444
	n (%)	
**Sex**		
Female	1,060 (42.2)	784 (27.5)
Male	1,454 (57.8)	2,062 (72.5)
Missing	294	787
**Principal Diagnosis**		
Injury & poisoning	633 (22.5)	3,206 (88.2)
Respiratory	581 (20.7)	13 (0.4)
Circulatory system	520 (18.5)	295 (8.1)
Neurological	371 (13.2)	67 (1.8)
Infectious & parasitic diseases	365 (13.0)	17 (0.5)
Other	337 (12.0)	35 (1.0)
Missing	1	0

Of the 2,808 retrieval taskings over the study period, 2,748 (97.9%) resulted in EMRS deployment to the patient. The remaining 60 taskings were stood down: 22 (36.6%) due to clinical deterioration (including patient death prior to team arrival, or a shift in focus to local end of life care), 20 (33.3%) due to adverse weather (with retrieval at a later date), 3 (5%) due to transfer by other means, 2 (3.3%) due to simultaneous retrievals elsewhere preventing transfer, and 1 (1.6%) due to clinical improvement; for 12 (20.0%) the reason was not recorded.

**Fig. 1:**
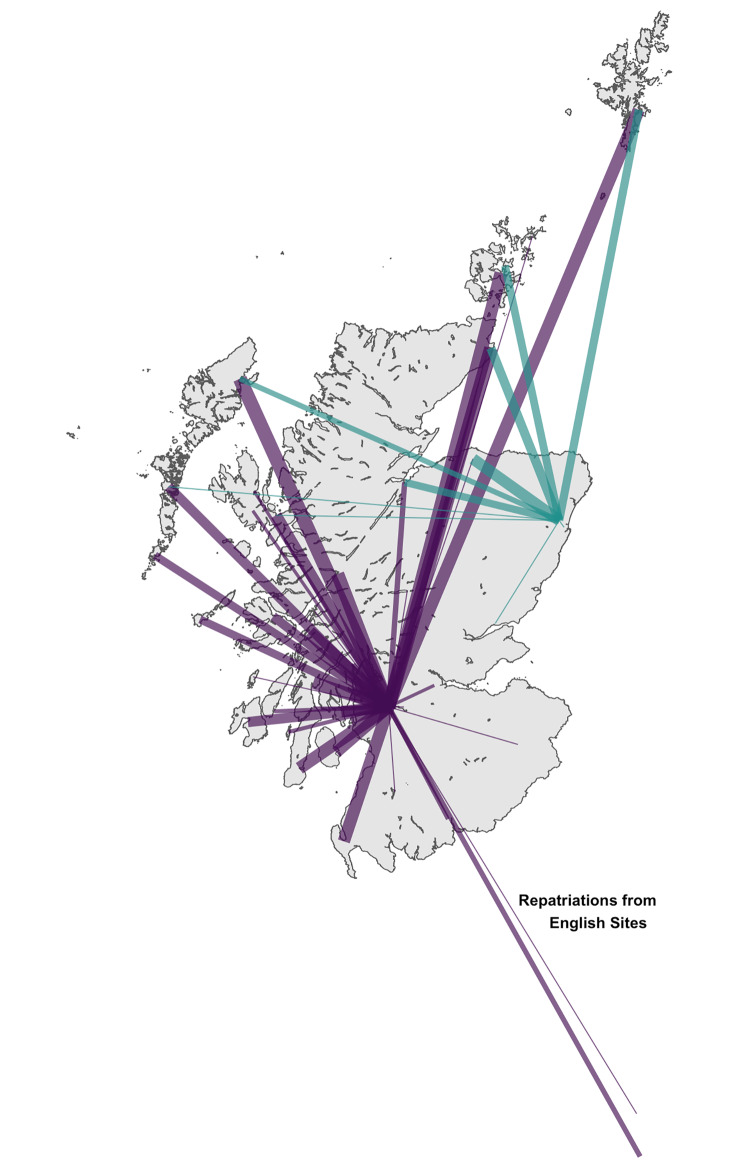
Map of referral locations for retrieval taskings to Emergency Medical Retrieval Service. Width of line from origin to referring centre is proportional to the number of retrievals undertaken to each destination

**Fig. 2:**
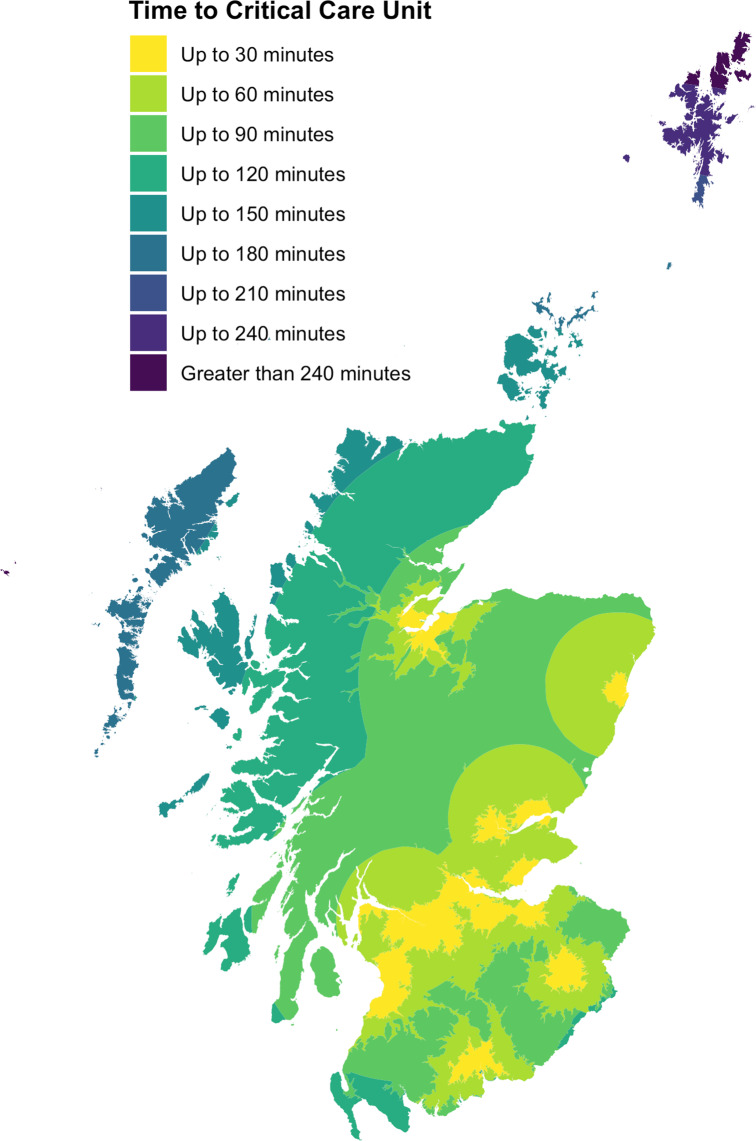
Isochrone maps of time to critical care unit in Scotland in two transport models. The first, modelled time by road alone to each critical care unit (Fig. 2**a**) and the second, modelled time using the faster of either road or the current configuration of aeromedical services to each critical care unit (Fig. 2**b**)

**Fig. 3:**
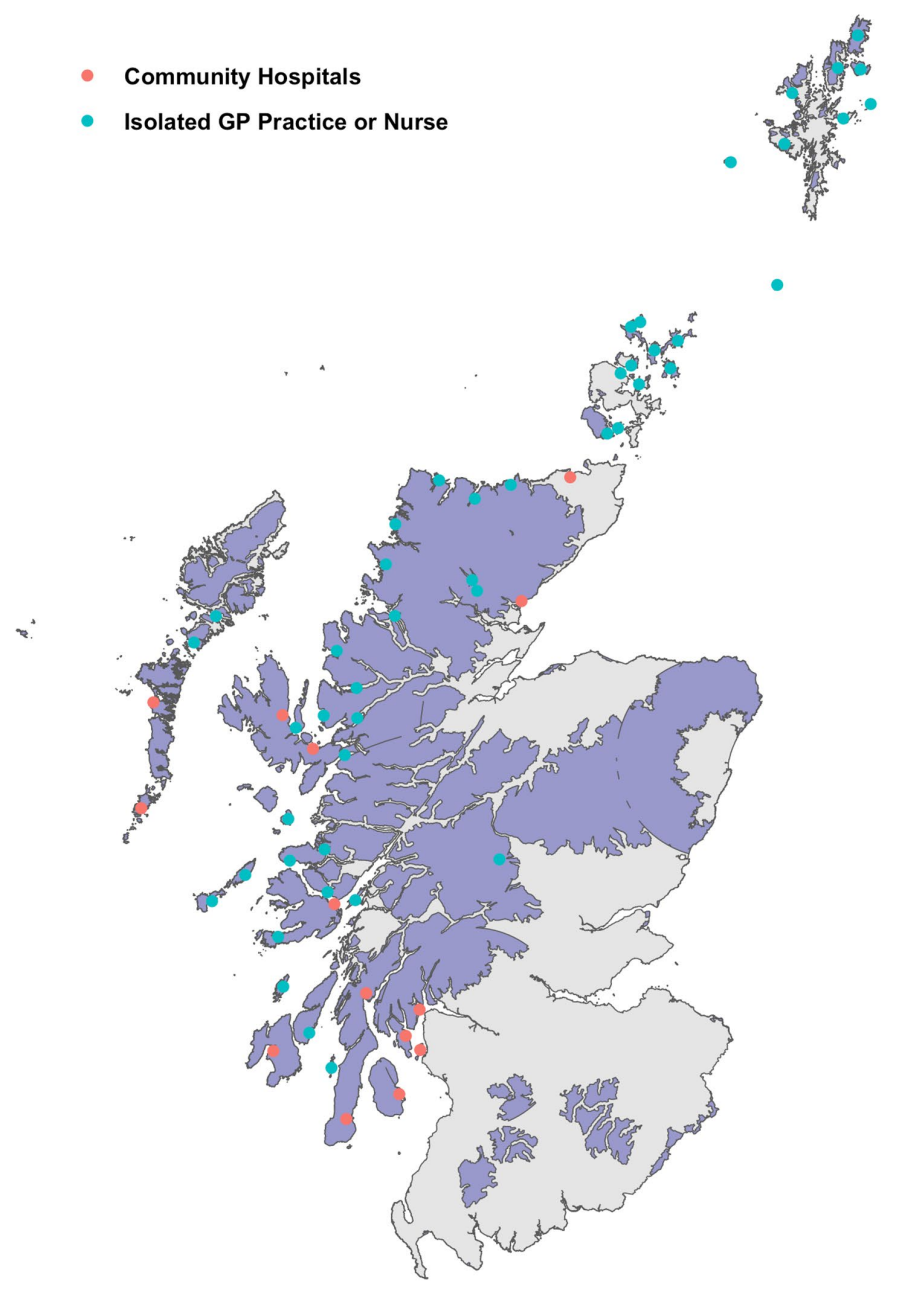
Map of Scotland, with shaded area indicating areas where EMRS provides fastest modelled access to initiation of critical care, with locations of remote community hospitals and isolated general practitioner or nursing provision

Retrieval missions were tasked to locations ranging from 3 to 552 km from EMRS bases (median distance 114 km). A map of retrieval taskings, showing origin of EMRS team, referral centre, and frequency of referral from each location is presented in Fig. 1. Isochrone maps were plotted, modelling time by road alone, and in combination with the current configuration of aeromedical services, to critical care units in Scotland (Fig. 2). The location of each referring centre was mapped to these modelled times, and the frequency of referrals from varying degrees of geographic isolation (as defined by time to critical care) are shown in Supplementary Fig. 2. The estimated mean modelled time to critical care unit by road for patients retrieved by EMRS was 3 h 31 min, reducing to 1 h 45 min using the model incorporating aeromedical transport. The maximum modelled time to critical care unit was 12 h by road (Gilbert Bain Hospital, Lerwick to Aberdeen Royal Infirmary), reducing to 3 h 45 min using the aeromedical model. The transport modalities used to reach patients are presented in Supplementary Fig. 3. The areas of Scotland where EMRS provides fastest modelled access to initiation of critical care is illustrated in Fig. 3, an area of 41,865km^2^ and approximate population of 213,409 based on 2011 census data.

Key physiological parameters for patients retrieved, at various points of their journey, are shown in Supplementary Table 1. A significant reduction in numbers of patients with systolic blood pressure < 90mmHg and respiratory rate outside of 6–30 per minute was observed between EMRS attendance and both departure from referring centre and arrival at destination hospital.

During transfer, 1,474 (52.5%) patients were mechanically ventilated and 1,675 (59.7%) received another critical care intervention such as pre-hospital blood transfusion, chest drain insertion, neuroprotective measures or point-of-care ultrasound scan.

### Pre-hospital critical care

**Fig. 4:**
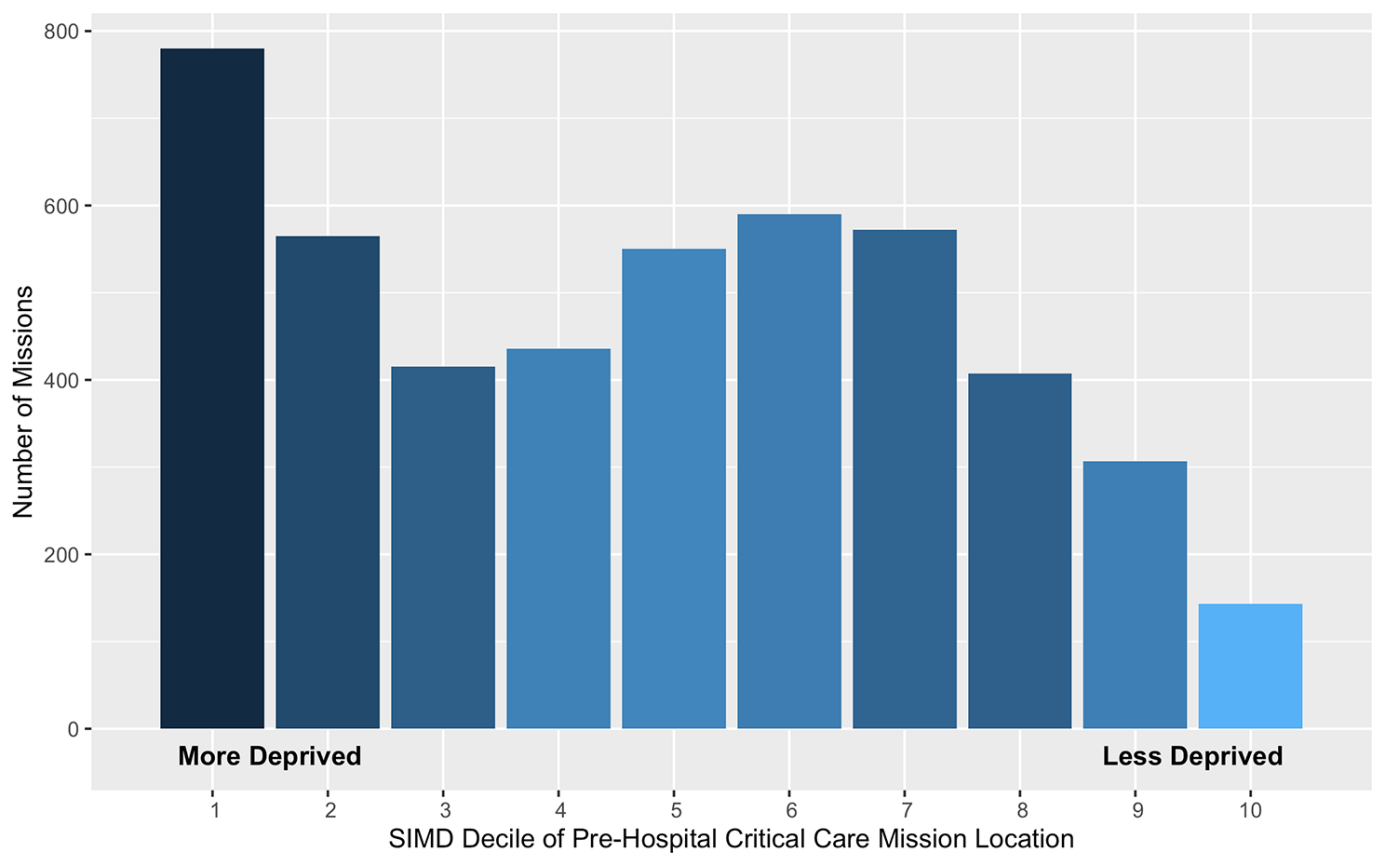
Bar chart of pre-hospital critical care mission frequency by Scottish Index of Multiple Deprivation decile

EMRS was tasked to 5,261 pre-hospital critical care missions. The team was stood down before clinical contact in 1,628 cases (30.9%) (Supplementary Fig. 4), resulting in 3,633 attendances. Of the 2,846 patients where sex was recorded, 784 (27.5%) were female. Of the 3,189 patients for whom age was recorded, 393 (12.3%) were under 18 years of age. The median age of patients receiving pre-hospital critical care was 42 (range 0–97) years. Including those where EMRS teams were ultimately stood down, 4,365 pre-hospital critical care taskings contained sufficient information to determine location. Distance from EMRS base to pre-hospital critical care location ranged from 0.2 to 327 km, with a median distance of 22 km (Supplementary Fig. 5). The number of missions varied by SIMD decile of mission location, with more missions tasked to more deprived areas (Spearman’s rank correlation, r(8)=-0.75, p = 0.01) (Fig. 4).

The majority of EMRS pre-hospital critical care activations were for trauma, including: 1,664 (45.8%) road traffic collisions, 320 (8.8%) interpersonal violence, 249 (6.9%) self-harm or suicidal events, and 102 (2.8%) occupational or industrial incidents. Medical events, such as cardiac arrest, accounted for 418 (11.5%), and 190 had no information on mechanism recorded.

Significant reductions in the proportion of patients with abnormal respiratory rate were seen as pre-hospital care progressed, while the numbers of patients with GCS ≤ 14 rose significantly (Supplementary Fig. 1). Advanced airway management was provided to 829 (22.8%) patients attended for pre-hospital critical care; of these, 761 (91.8%) received tracheal intubation, 60 (7.2%) a supraglottic airway device, and 8 (1.0%) a surgical airway. Other critical care interventions (as above) were delivered to 914 (25.2%) patients. Transport modalities used to reach patients are presented in Supplementary Fig. 3.

Overall, 345 (9.5%) of 3,633 pre-hospital critical care attendances resulted in pre-hospital death. On univariate analysis, pre-hospital death was more likely among patients who were older, had low GCS or systolic blood pressure, or respiratory rate outside the normal range on EMRS arrival (Table 2).

**Table 2:** Binary logistic regression analysis of the demographic and physiological factors associated with pre-hospital mortality among pre-hospital critical care missions

		Univariate analysis		Adjusted for demographic and physiological factors	
		Estimated OR (95% CI)	p value	Estimated OR (95% CI)	p value
Sex	Male	Reference		Reference	
	Female	0.81 (0.55–1.17)	0.24	0.50 (0.10–2.56)	0.34
Age (years)	0–16	1.15 (0.59–2.25)	0.68	0.55 (0.12–2.42)	0.41
	17–24	Reference		Reference	
	25–39	1.10 (0.61–1.98)	0.75	1.37 (0.20–9.47)	0.72
	40–64	1.70 (0.95–3.07)	0.07	1.33 (0.35–5.08)	0.66
	**≥ 65**	**2.21 (1.15–4.26)**	**0.02**	1.87 (0.32–10.78)	0.45
Distance from EMRS Base (km)	0–10	Reference		Reference	
	10–20	**0.72 (0.52–0.99)**	**0.04**	0.93 (0.35–2.43)	0.87
	20–40	0.80 (0.57–1.12)	0.19	0.84 (0.28–2.53)	0.75
	40–100	0.88 (0.62–1.23)	0.44	1.75 (0.33–9.27)	0.46
	> 100	0.66 (0.43–1.02)	0.06	0.76 (0.19–2.97)	0.67
Observations on EMRS Arrival					
Systolic Blood Pressure (mmHg)	≥ 90mmHg	Reference		Reference	
	< 90mmHg	**1423.48 (69.93- 28975.63)**	**< 0.01**	**469.93 (37.98- 5813.96)**	**< 0.01**
Respiratory Rate (Breaths per Minute)	6–30	Reference		Reference	
	< 6 or > 30	**142.25 (61.41- 329.51)**	**< 0.01**	**28.76 (13.58–60.90)**	**< 0.01**
Glasgow Coma Score	15	Reference		Reference	
	≤ 14	**167.70 (38.13- 737.53)**	**< 0.01**	**16.94 (4.81–59.69)**	**< 0.01**

There was a U-shaped relationship with distance from base, with pre-hospital death more common for short and long distances. In the multivariate model, only the associations with physiological measurements remained statistically significant.

## Discussion

Our study demonstrated a considerable expansion of EMRS workload over the studied ten year period; with a greater than twenty-fold increase in pre-hospital critical care taskings, and widening of the range of patient ages, distances travelled and diseases treated compared to previous work describing the origins of the service [1]. Geospatial modelling suggested that use of aeromedical transportation reduced the mean time for retrieval to critical care unit by 1 h 46 min, and in some cases by up to 8 h, compared with road/ferry transportation; and that EMRS provides fastest access to critical care initiation for the majority of Scottish land mass and a substantial population. As isolation has the potential to adversely affect health outcomes [16], these findings reinforce that aeromedical critical care has a role in delivering equitable healthcare, while preserving local resource that may otherwise be diverted to prolonged patient transfer. Pre-hospital critical care may play a role in mitigating health inequalities due to higher rates of dispatch to socioeconomically deprived areas known to suffer greater disease burden, particularly in substance misuse disorders, penetrating trauma, interpersonal violence and self-harm [17–19]. These findings were consistent with a study of retrieval services in Western Australia that reported higher rates of aeromedical retrieval among aboriginal patients, a group experiencing socioeconomic disadvantage [20].

The sex breakdown of patients referred for retrieval is similar to that described in critical care audit projects [21]. The male preponderance of patients receiving pre-hospital critical care is consistent with the higher risk of major trauma among young men described in the literature and national audit [22, 23]. However, there is increasing awareness that trauma systems may respond differently to men and women, for example in the receipt of evidence-based management [24], and that major trauma in older populations may be under-triaged in pre-hospital care [25, 26].

Consistent with previous studies [22], abnormal physiological measurement were significantly associated with mortality. However, our study demonstrated improvements in most of these parameters while in the care of EMRS. Whilst the reduction in GCS score through the pre-hospital phase of care may reflect deterioration in clinical condition, it could also be explained by use of pre-hospital sedation or anaesthesia. As predictors of both increased risk and greater potential to benefit, abnormal physiological measurements should be important criteria for pre-hospital and retrieval team tasking.

Previous studies have suggested higher mortality among older patients [22, 27, 28], although did not report pre-hospital deaths, suggesting potential survival bias. In contrast, our study demonstrated that while older age was associated with pre-hospital mortality univariately, it was no longer associated following adjustment for potential physiological confounders. This suggests that age, in isolation, should not be a criterion for activation of pre-hospital critical care.

A U-shaped relationship was demonstrated between distance from EMRS base and pre-hospital mortality on univariate analysis. Higher mortality following short distance taskings may be explained by a higher clinical acuity in nearby taskings whereas more stable patients may be transported quickly to hospital without pre-hospital critical care response. Additionally, shorter travel times allow attendance in instances where patients may otherwise have life pronounced extinct by local clinicians. The validity of these potential mechanisms was corroborated by the finding that these differences in mortality lost significance following adjustment for physiological measurements. Further work is required to explore these associations and mechanisms.

The COVID-19 pandemic created sustained challenges for the delivery of healthcare and the recalibration of healthcare delivery is seen in the concomitant reduction in retrieval taskings. Disruption to normal activities, particularly reduced road traffic arising from mitigation measures instituted early in the pandemic [29], is thought to have contributed to the reduction in pre-hospital critical care undertaken in 2020.

This study benefits from deriving its data sources from a comprehensive database spanning the study period and contemporaneous input, minimising reporting and recall bias respectively. Geolocation ensured accurate mapping of pre-hospital critical care missions. However, this work also has limitations. Transcription of paper records to a computerised database, even by service clinicians, may introduce errors, and the nature of data collection has changed over time, resulting in incomplete data across some variables. Multiple imputation methods address confounding that may be introduced when using complete-case analysis, but has limitations compared to complete data [30]. Diagnosis in pre-hospital environments may be challenging [31], and mapping initially-recorded diagnoses to ICD-10 principal diagnosis may introduce error. Modelled time to critical care assumes immediate dispatch capability, and does not take into account variation by time of day, or limitations such as weather. Pre-hospital critical care missions were mapped to areas of each decile of the Scottish Index of Multiple Deprivation by location, not patient home address, which may introduce confounders in determining the socioeconomic characteristics of individual patients attended by the service. While this study reports the Scottish experience, it is not known to what degree these findings may be replicated in other contexts internationally given variation in the provision of pre-hospital critical care. Findings such as that of more frequent tasking to areas of socioeconomic deprivation, or our quantification of the utility of an aeromedical model in areas of geographic isolation, are likely to be generalisable on the basis of related literature [19, 20]; however others, such as the growth in case numbers over time, are likely influenced by local service expansion.

It is important to note that, while EMRS is the only service providing aeromedical pre-hospital critical care in Scotland, this study does not consider other services providing enhanced pre-hospital care, including Medic One in Edinburgh, Tayside Trauma Team in Dundee, Highland Pre-Hospital Immediate Care & Trauma based in Inverness, Scottish Ambulance Service Advanced Practitioners in Critical Care, and BASICS clinicians across remote and rural Scotland. The work of the Air Ambulance Service, and ScotSTAR paediatric and neonatal retrieval teams are also not considered in this work.

## Conclusions

Pre-Hospital and Retrieval Medicine in Scotland has evolved from its inception in 2004, and over its first 10 years as a national service. More patients, over a greater range of ages, with more diverse pathologies are being treated and transported by the service.

As other services, including the Scottish Trauma Network and Care of Burns in Scotland network, increasingly emphasise a whole system, centralised approach to care, the requirement to treat critically ill patients close to the site of illness or injury, and transport them safely to the best place of care, remains a key link in the chain of survival. This work on the Scottish model of Pre-Hospital and Retrieval Medicine, and the outcomes of the patients it serves, demonstrates the value of aeromedical and critical care retrieval for the initiation and development of similar services internationally.

### Electronic supplementary material

Below is the link to the electronic supplementary material.

Supplementary Material 1

## Data Availability

The datasets analysed during the current study are available from the corresponding author on reasonable request.

## Abbreviations

EMRS: Emergency Medical Retrieval Service
ScotSTAR: Scottish Specialist Transport and Retrieval
SAS: Scottish Ambulance Service
SCAA: Scotland’s Charity Air Ambulance
SAR: Search and Rescue
GCS: Glasgow Coma Score
GP: General Practitioner
