# Supplementary material for: Pre-hospital and retrieval medicine in Scotland: a retrospective cohort study of the workload and outcomes of the emergency medical retrieval service in the first decade of national coverage
Source: Scand J Trauma Resusc Emerg Med. 2023 Aug 22;31:39. doi: 10.1186/s13049-023-01109-6 (PMC10463457; doi:10.1186/s13049-023-01109-6)
Supplement: Supplementary file 1 — Supplementary Material 1 [file 13049_2023_1109_MOESM1_ESM.docx]

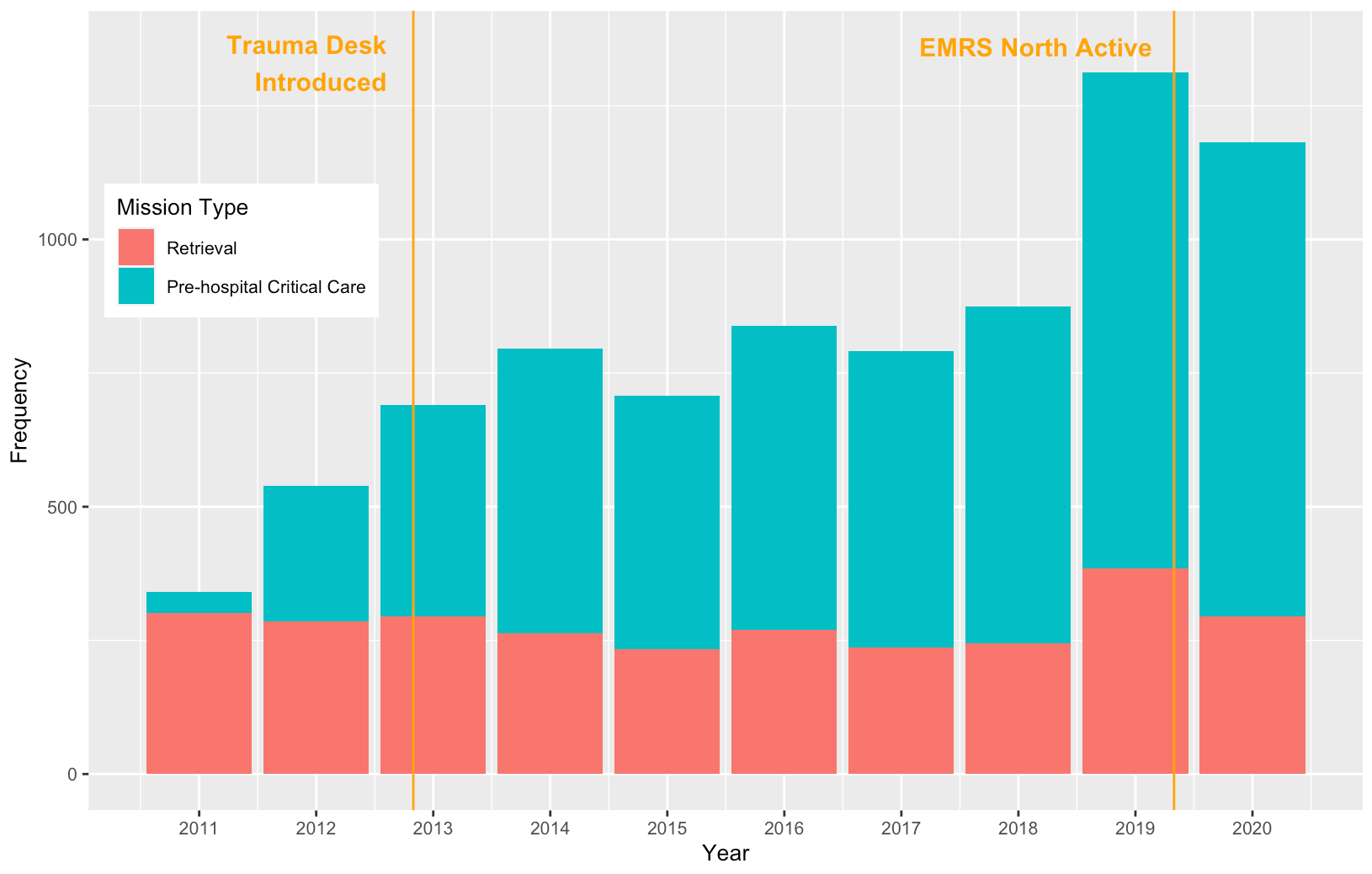
Supplementary Figure 1. Bar chart of number of Emergency Medical Retrieval Service taskings by mission type 2011-2020. Key events in the development of the service are highlighted.

|  | Systolic blood pressure measurements | | | | Glasgow Coma Scale observations | | | | Respiratory rate observations | | | |
| --- | --- | --- | --- | --- | --- | --- | --- | --- | --- | --- | --- | --- |
|  | Total  n | <90mmHg  n (%) | OR (95% CI) | p value | Total  n | ≤14  n (%) | OR (95% CI) | p value | Total  n | <6 or >30  n (%) | OR (95% CI) | p value |
| **Retrieval Missions** | | | | | | | | | | | | |
| At EMRS Arrival | 2445 | 272 (11.1) | Reference | | 2459 | 1562 (63.4) | Reference | | 2410 | 155 (6.4) | Reference | |
| At EMRS Departure | 2390 | 105 (4.4) | 0.37 (0.29-0.46) | <0.01 | 2404 | 1575 (65.5) | 1.09 (0.97-1.23) | 0.15 | 2420 | 89 (3.7) | 0.56 (0.43-0.73) | <0.01 |
| On Arrival to Receiving Centre | 2294 | 100 (4.4) | 0.36 (0.29-0.46) | <0.01 | 2296 | 1475 (64.2) | 1.03 (0.92-1.16) | 0.61 | 2295 | 85 (3.7) | 0.56 (0.43-0.73) | <0.01 |
| **Pre-Hospital Critical Care Missions** | | | | | | | | | | | | |
| At EMRS Arrival | 2175 | 178 (8.2) | Reference | | 2864 | 1177 (41.1) | Reference | | 2707 | 352 (13) | Reference | |
| At EMRS Departure | 1701 | 138 (8.1) | 0.99 (0.79-1.25) | 0.94 | 2109 | 985 (46.7) | 1.26 (1.12-1.41) | <0.01 | 2031 | 118 (5.8) | 0.41 (0.33-0.51) | <0.01 |
| On Arrival to Receiving Centre | 1552 | 108 (7.0) | 0.84 (0.65-1.08) | 0.17 | 1732 | 865 (49.9) | 1.43 (1.27-1.61) | <0.01 | 1690 | 72 (4.3) | 0.30 (0.23-0.39) | <0.01 |

Supplementary Table 1. Physiological parameters of patients attended by the Emergency Medical Retrieval Service, stratified by phase of care and mission type with univariate logistic regression of change through phases of care.


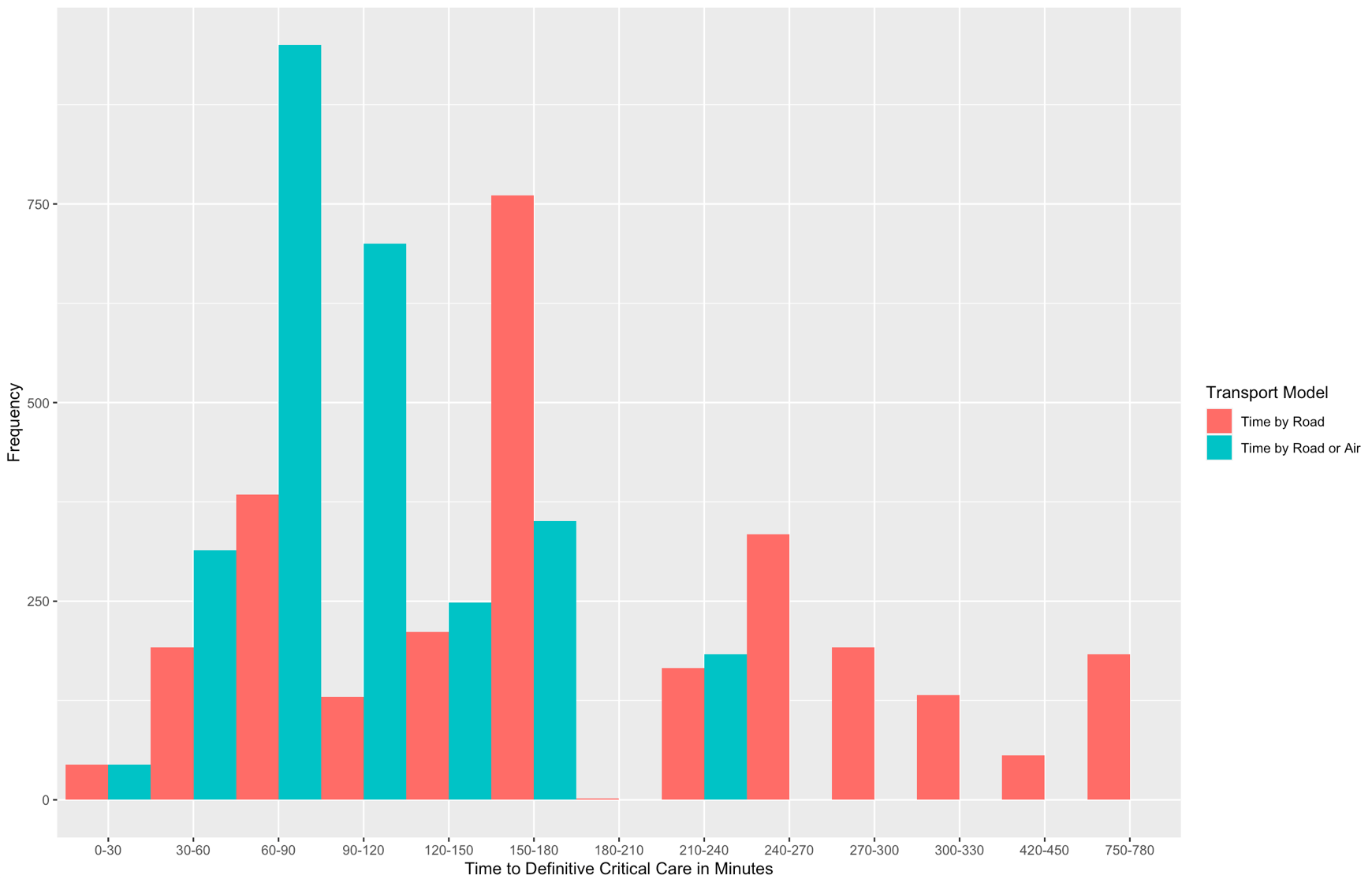
Supplementary Figure 2. Bar plot of Emergency Medical Retrieval Service retrieval missions by time to critical care unit, modelled by road alone, and modelled by the faster of either road or aeromedical transport.

| **Modelled Time to Critical Care Unit (minutes)** | Number of patients (%) | |
| --- | --- | --- |
|  | Road Model | Road and Aeromedical Model |
| 0-30 | 44 (1.6%) | 44 (1.6%) |
| 31-60 | 192 (6.9%) | 314 (11.3%) |
| 61-90 | 384 (13.8%) | 947 (34.0%) |
| 91-120 | 130 (4.7%) | 700 (25.1%) |
| 121-150 | 211 (7.6%) | 248 (8.9%) |
| 151-180 | 761 (17.3%) | 351 (12.6%) |
| 181-210 | 2 (<0.1%) | 0 |
| 211-240 | 166 (6.0%) | 183 (6.6%) |
| ≥241 | 897 (32.2%) | 0 |
| Missing Location | 21 | 21 |

Supplementary Figure 3. Bar plot of Emergency Medical Retrieval Service transport modalities by mission type.


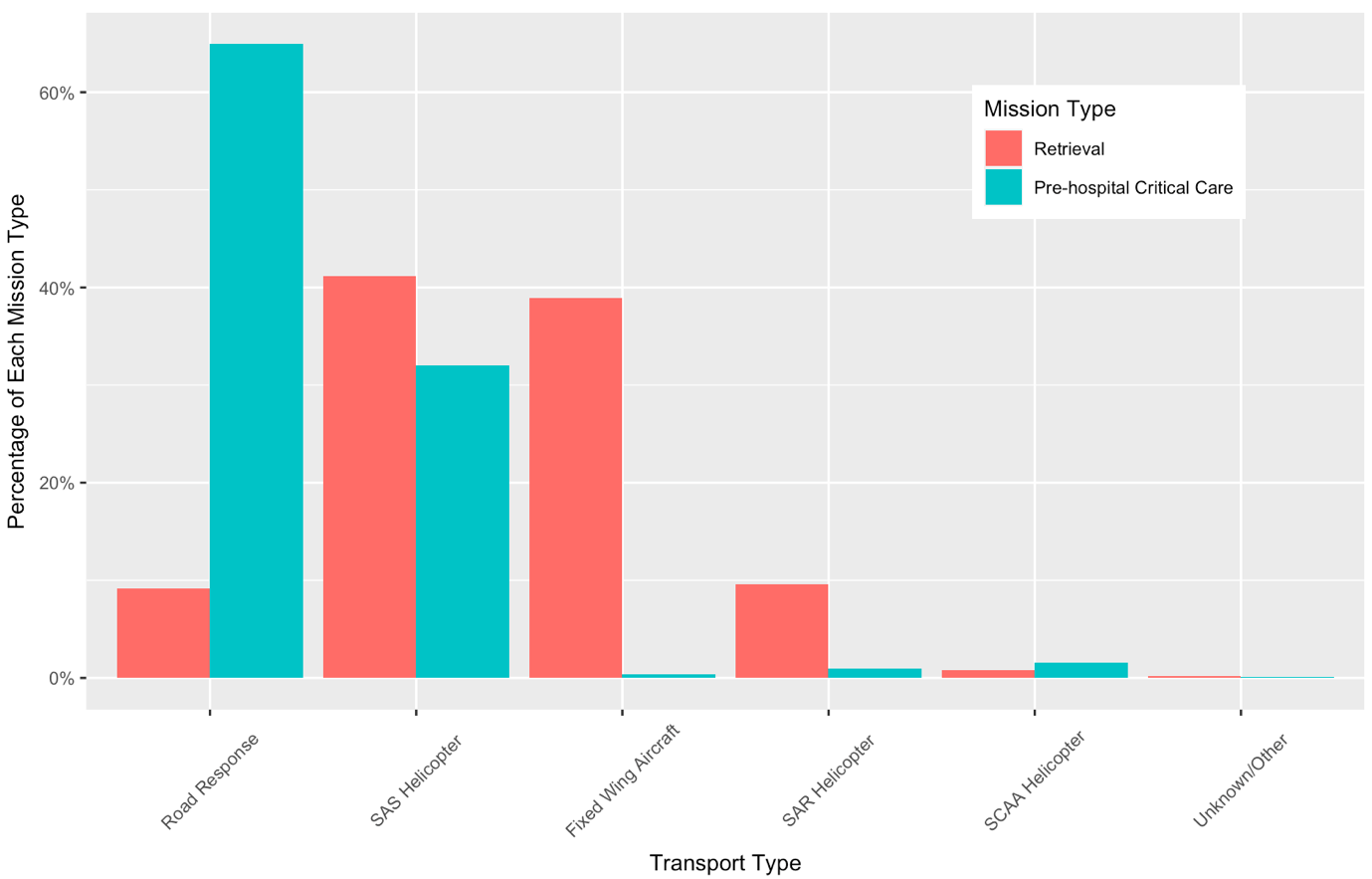


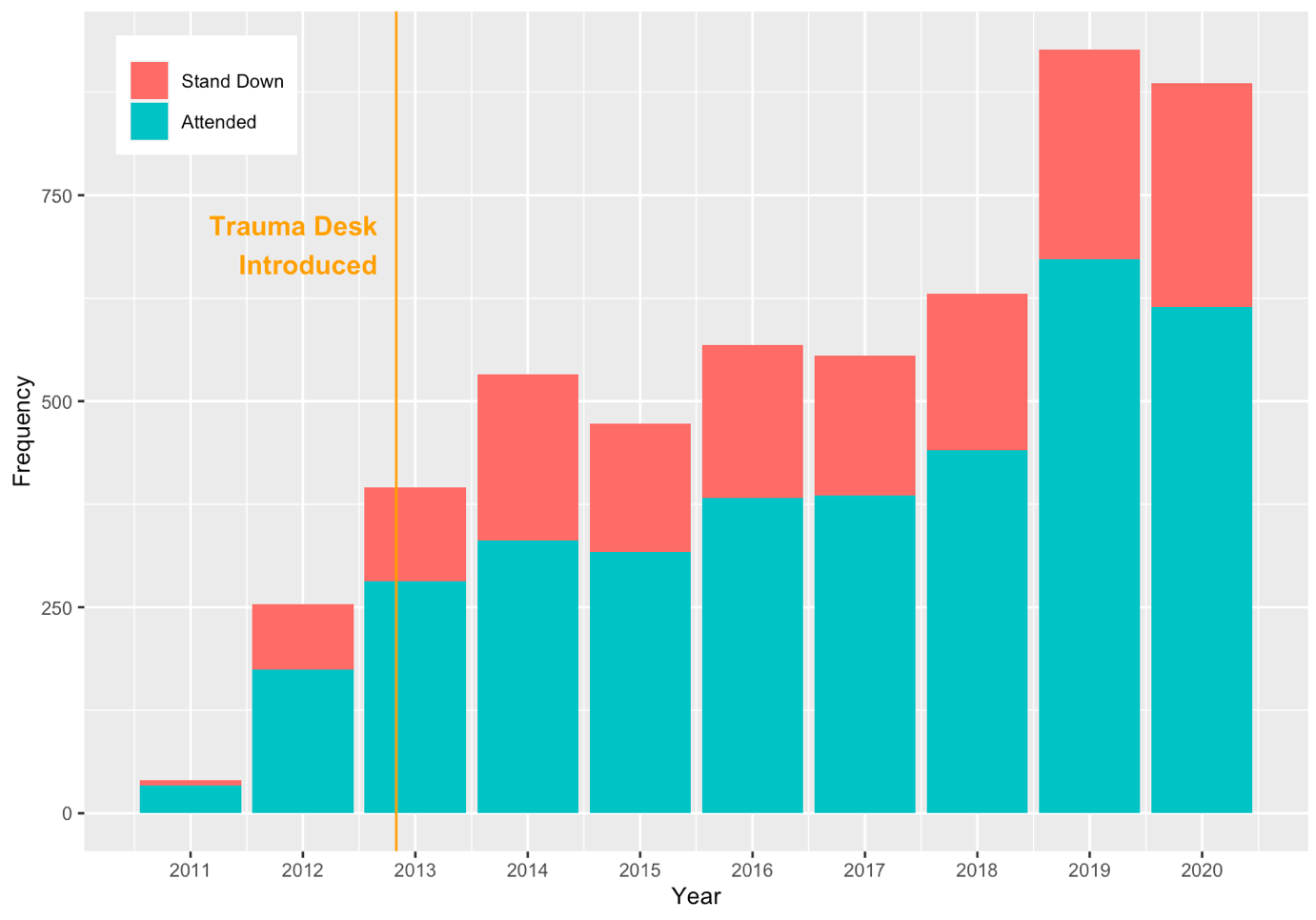
Supplementary Figure 4. Attendance and stand down rate for Emergency Medical Retrieval Service pre-hospital critical care missions by year

Supplementary Figure 5. Map of geographic distribution of Emergency Medical Retrieval Service pre-hospital critical care missions 2011-2020
